# Potential long-term treatment of hemophilia A by neonatal co-transplantation of cord blood-derived endothelial colony-forming cells and placental mesenchymal stromal cells

**DOI:** 10.1186/s13287-019-1138-8

**Published:** 2019-01-22

**Authors:** Kewa Gao, Priyadarsini Kumar, Elizabeth Cortez-Toledo, Dake Hao, Lizette Reynaga, Melanie Rose, Chuwang Wang, Diana Farmer, Jan Nolta, Jianda Zhou, Ping Zhou, Aijun Wang

**Affiliations:** 1 Department of Burns and Plastic Surgery, The Third Xiangya Hospital of Central South University, Changsha, Hunan 410013 People’s Republic of China; 2 Surgical Bioengineering Laboratory, Department of Surgery, University of California Davis, Sacramento, CA 95817 USA; 3 Institute for Pediatric Regenerative Medicine, Shriners Hospitals for Children, Northern California, Sacramento, CA 95817 USA; 4 Department of Internal Medicine, Stem Cell Program and Institute for Regenerative Cures, University of California Davis, Sacramento, CA 95817 USA; 5 Department of Biomedical Engineering, University of California Davis, Davis, CA 95616 USA

**Keywords:** Hemophilia A, Mesenchymal stromal cells (MSCs), Endothelial colony-forming cells (ECFCs), Cell engraftment, Co-transplantation, Neonatal

## Abstract

**Background:**

Hemophilia A (HA) is an X-linked recessive disorder caused by mutations in the Factor VIII (FVIII) gene leading to deficient blood coagulation. As a monogenic disorder, HA is an ideal target for cell-based gene therapy, but successful treatment has been hampered by insufficient engraftment of potential therapeutic cells.

**Methods:**

In this study, we sought to determine whether co-transplantation of endothelial colony-forming cells (ECFCs) and placenta-derived mesenchymal stromal cells (PMSCs) can achieve long-term engraftment and FVIII expression. ECFCs and PMSCs were transduced with a B domain deleted factor VIII (BDD-FVIII) expressing lentiviral vector and luciferase, green fluorescent protein or Td-Tomato containing lentiviral tracking vectors. They were transplanted intramuscularly into neonatal or adult immunodeficient mice.

**Results:**

In vivo bioluminescence imaging showed that the ECFC only and the co-transplantation groups but not the PMSCs only group achieved long-term engraftment for at least 26 weeks, and the co-transplantation group showed a higher engraftment than the ECFC only group at 16 and 20 weeks post-transplantation. In addition, cell transplantation at the neonatal age achieved higher engraftment than at the adult age. Immunohistochemical analyses further showed that the engrafted ECFCs expressed FVIII, maintained endothelial phenotype, and generated functional vasculature. Next, co-transplantation of ECFCs and PMSCs into *F8* knock-out HA mice reduced the blood loss volume from 562.13 ± 19.84 μl to 155.78 ± 44.93 μl in a tail-clip assay.

**Conclusions:**

This work demonstrated that co-transplantation of ECFCs with PMSCs at the neonatal age is a potential strategy to achieve stable, long-term engraftment, and thus holds great promise for cell-based treatment of HA.

**Electronic supplementary material:**

The online version of this article (10.1186/s13287-019-1138-8) contains supplementary material, which is available to authorized users.

## Background

Hemophilia A (HA) is an X-link recessive coagulation disorder caused by lack of coagulation factor VIII (FVIII) with an incidence of 1 in 5000 male births [1]. Patients with severe HA, defined as FVIII activity less than 1% of normal, suffer from debilitating hemarthrosis, life-threatening internal bleeding, and potentially fatal intracranial hemorrhages [2]. Currently, the standard of care for these patients is FVIII protein substitution therapy (PST) with repeated intravenous injection of the FVIII concentrates derived from human plasma or the FVIII protein generated through recombinant DNA technology [3, 4]. While this protein-based treatment has greatly improved the quality of life and extended the life expectancy of many HA patients, its high cost and the need for lifelong infusion makes it far from an ideal therapy [5]. Furthermore, patients under PST still experienced bleeding episodes because of the fluctuation of the infused proteins. Novel therapies to achieve sustained FVIII expression in HA patients are needed to overcome the serious limitations of current treatments.

As a monogenic disorder, HA is considered a highly attractive target for gene therapy [6, 7], and even a 3–5% increase in FVIII levels can substantially reduce symptoms and alleviate severe HA phenotype to moderate HA phenotype [8]. A recent landmark clinical trial in hemophilia A showed that an adeno-associated virus serotype 5 (AAV5) vector-based gene therapy achieved therapeutic levels of FVIII expression in the patients over 1 year [9, 10]. But long-term effects of the AAV-based gene therapy are yet to be determined. In addition, about one third of HA patients are not suitable candidates for this therapy because of their previous exposure to AAV [11]. Ex vivo stem cell-based gene therapy avoids direct administration of a large quantity of the viral vector and circumvents the drawbacks of an immune response to the viral vector. Since endothelial cells (ECs) are the primary source of FVIII in the body [12, 13], a number of studies have shown that ECs and endothelial progenitor cells (EPCs) can correct the phenotype in HA animal models [14–21]. However, primary ECs, such as liver sinusoidal ECs, are often limited by their availability. Endothelial colony-forming cells (ECFCs) are a subset of circulating EPCs in the peripheral or umbilical cord blood. Once isolated in culture, ECFCs are highly proliferative and possess all phenotypical and functional characteristics of ECs [22], and several studies have demonstrated their potential use in cell-based therapies.

Mesenchymal stromal cells (MSCs) derived from adult or perinatal tissues have been well established as a stem cell therapy product for a wide variety of diseases and conditions. Placenta-derived MSCs (PMSCs) represent an emerging and exciting therapeutic agent for disease treatment. The placenta is a unique, fetal-derived tissue [23], and a number of studies including our own preclinical research have shown that PMSCs can reliably be obtained from early gestation placenta and used for the treatment of developmental and perinatal diseases [24–26]. These early gestation PMSCs display notable immunomodulatory capabilities [27–29] and exhibit a greater capacity to improve wound healing [30] and ex vivo expansion potential compared to term placenta or adult bone marrow-derived mesenchymal stromal cells (BM-MSCs) [28, 31]. PMSCs can also secrete a variety of cytokines such as hepatocyte growth factor (HGF) and vascular endothelial growth factor (VEGF) that are critical for endothelial cells growth and functional angiogenesis. The in vivo localization of PMSCs is yet to be characterized. Whether all MSCs are pericytes that are perivascular cells is still debatable. Nevertheless, pericytes are important for endothelial cell protection and functional structure formation [32, 33]. Even though MSCs do not engraft long term after transplantation, their various functions make them an ideal cell type for co-transplantation applications [34–37]. It has been demonstrated that when ECFCs are in contact with MSCs, ECFCs secrete growth factors such as platelet-derived growth factor-BB (PDGF-BB) and fibroblast growth factor 2 (FGF-2) that improve MSC proliferation [38]. When ECFCs were co-transplanted with BM-MSCs, the ECFC engraftment rate was improved by interactions between ECFCs and MSCs through the NOTCH signaling pathway [39].

The interaction between the transplanted cells and the microenvironment at the site of transplantation can also significantly influence cell fate and its engraftment potential. To improve cell engraftment for the treatment of HA, previous studies transplanted liver sinusoidal endothelial cells (LSECs) to adult mouse models with liver endothelium injured by use of toxins such as monocrotaline (MCT) [18] or uPA [21] so that the transplanted cells are exposed to an environment where ECs are needed for the repair of endothelium. However, in clinical situations, these invasive methods pose unnecessary risks to patients and will not be feasible. Since the neonatal period is the fastest growing and developing period of life, ECFCs transplanted during this period of time could potentially be benefited from the developing niche and contribute to vascular expansion [40]. Hence, application of a cellular gene therapy to newborn HA patients could rescue the phenotype before advancement of the disease.

In this study, we intend to develop a novel cell-based gene therapy for the treatment of HA by co-transplantation of ECFCs and PMSCs. To achieve a high level of functional FVIII secretion, ECFCs and PMSCs were both transduced with a BDD-FVIII expressing lentiviral vector. We hypothesize that co-transplanting genetically modified ECFCs with PMSCs will achieve stable long-term engraftment and provide a phenotypic correction of the disease. We transplanted ECFCs and PMSCs alone or in combination, into immune-deficient mice, and compared their long-term cell engraftment. To identify the best age for the transplantation treatment, we also compared the engraftment of ECFCs and PMSCs in newborn and adult mice and assessed their engraftment. Finally, we evaluated whether co-transplanting genetically modified ECFCs with PMSCs alleviates the symptom of HA in an animal model.

## Methods

### Cell isolation and expansion

Discarded term placenta was obtained from the University of California Medical Center, and ECFCs were isolated from human umbilical cord blood as previously described [41]. Mononuclear cells (MNCs) that were obtained using Ficoll (GE Healthcare) gradient centrifugation were seeded onto rat-tail collagen type I (BD Biosciences Discovery) coated tissue culture dishes and cultured in Endothelial Cell Growth Medium MV-2 media (ECGM-MV2, PromoCell). ECFCs were used between passages 3 and 5 for all the experiments described in this study.

PMSCs were isolated from discarded first-trimester gestation placental tissue (gestation age of 11 weeks) collected at the University of California, Davis Medical Center, by using our established explant culture method [29]. PMSCs were cultured in media consisting of DMEM high glucose with 10% fetal bovine serum (FBS, Hyclone), and 100 U/ml penicillin (ThermoFisher Scientific) and 100 μg/ml streptomycin (ThermoFisher Scientific). PMSCs were used between passages 4 and 7 in all our experiments in this study.

### Lentiviral vector transduction

All lentiviral constructs were generated at the UC Davis Institute for Regenerative Cures (IRC) Vector Core. ECFCs and PMSCs alone groups were transduced with the pCCLc-MNDU3-LUC-PGK-EGFP-WPRE vector as well as the pCCLc-MNDU3-BBD F8-PGK-NEO-WPRE vector. To distinguish between ECFCs and PMSCs in the co-transplantation group, a Td-Tomato containing lentiviral vector pCCLc-MNDU3-F8-PGK-Tomato-WPRE was used to label PMSCs in the group. Transductions were performed in transduction media consisting of DMEM high glucose, 10% FBS, and 8 μg/ml protamine sulfate (MP Biomedicals) for 6 h. All vectors were transduced at a multiplicity of infection (MOI) of 10. After that, cells were cultured in ECGM-MV2 media for 72 h. After 72 h, cells were screened for neomycin resistance for 7 days cultured in media containing 2 μg/ml of G418 (EMD, Millipore). Cells were then cultured and expanded in ECGM-MV2 medium.

### Flow cytometry

The surface markers of ECFCs and PMSCs were characterized by flow cytometry. All antibodies were obtained from BD Biosciences. ECFCs were stained with APC-CD45 (560973), Alexa Fluor 647-CD31 (561654), PE-CD34 (550761), APC-CD105 (562408), PE-CD144 (561714), PE-CD14 (561707), Alexa Fluor 647-CD309 (560495), and PE-CD146 (550315) while PMSCs were stained with APC-CD29 (561794), FITC-CD44 (560977), APC-CD73 (560847), FITC-CD31 (560984), FITC-CD90 (561969), FITC-CD34 (560942), APC-CD45 (560973), and APC-CD105 (562408). APC-Ms IgG1 κ (550854), FITC-Ms IgG2b κ (556655), FITC-Ms IgG1 κ (556650), and Alexa Fluor 647-Ms IgG1 κ (557783) were used as isotype controls, and anti-mouse Igκ CompBeads were used to generate compensation controls. Transduction efficiency was assessed by GFP or Td-Tomato expression. The Attune NxT Flow Cytometer (ThermoFisher Scientific) was used for performing flow cytometry, and FlowJo software (FlowJo LLC) was used for data analyzing.

### Acetylated low-density lipoprotein uptake

ECFCs were cultured in 0.5% BSA for 24 h and were then incubated with 10 μg/ml Dil-AcLDL (Alfa Aesar) in serum-free culture medium for 5 h at 37 °C. Cells were then washed three times with PBS and fixed with 10% formalin for 15 min and stained with DAPI (1:5000 in water) to label the nuclei. The cells were imaged with a Zeiss Observer Z1 microscope.

### Tube formation assay

Twenty-four-well culture dishes were coated with 300 μl Matrigel (BD Biosciences) per well and allowed to gel for 60 min at 37 °C. 1 × 10^5^ ECFCs were seeded onto the Matrigel-coated wells and incubated at 37 °C, 5% CO_2_. Phase contrast images were taken at 6 and 16 h after seeding using Zeiss Observer Z1 microscope.

### Enzyme-linked immunosorbent assay (ELISA)

ECFCs were seeded in a 6-well culture dish at 5 × 10^5^ cells per well and cultured in 2 ml of ECGM-MV2 and incubated at 37 °C, 5% CO_2_. After 48 h, we collected the conditioned media and removed cell debris by centrifugation at 1500 rpm for 10 min. Quantification of FVIII protein secreted by cells present in undiluted conditioned media was assessed using FVIII ELISA kit (Affinity Biologicals) as per the manufacturer’s instructions. The standard was generated by human calibrator plasma (Affinity Biologicals).

### Chromogenic assay

FVIII activity in undiluted conditioned media was prepared as described above for FVIII ELISA which was performed using Coamatic FVIII kit (Chromogenix) as per the manufacturer’s instructions. The standard was generated by coagulation reference calibration plasma (Technoclone).

### RT-PCR

Total RNA was extracted from cells or tissue using the RNeasyPlus Mini kit (Qiagen), and cDNA was synthesized using Superscript II Reverse transcriptase (ThermoFisher Scientific). We amplified target genes using AccuPower PCR premix (Bioneer) at an annealing temperature of 60 °C for 35 cycles, and products were analyzed in a 2% agarose gel. The primer sequences of human GAPDH and BDD-F8 are shown in Additional file 1: Table S1.

### In vivo cell transplantation

All animal procedures were approved by The University of California, Davis (UCD) institutional animal care and use committee (IACUC). All facilities used during the study period were accredited by the Association for the Assessment and Accreditation of Laboratory Animal Care International (AAALAC). NSG (NOD/SCID/IL2Rγ^−/−^) immunodeficient mice were purchased from The Jackson Laboratory. Cells were resuspended in 16.7 μl ECGM-MV2 media and 3.3 μl of Matrigel (BD Biosciences) and injected intramuscularly to the left hind limb of each mouse. 3 × 10^5^ GFP/LUC-labeled ECFCs or PMSCs were used for ECFC only group and PMSC only group, respectively. For the co-transplantation group, 3 × 10^5^ GFP/LUC-labeled ECFCs and 2 × 10^5^ Td-Tomato-labeled PMSCs were used. For the neonatal group, cells were transplanted to mice at 3–5 days of age, and for the adult group, cells were transplanted at 12 weeks of age.

To test the efficacy of the cells in the disease model of HA, F8 mutant HA mice (B6;129S-F8^tm1Kaz^/J) were purchased from The Jackson Laboratory. To avoid immune rejection of the transplanted human cells in the immunocompetent HA mice, 210 mg/ml cyclosporine A was added to the drinking water. 3 × 10^6^ GFP/LUC-labeled ECFCs and 2 × 10^6^ Td-Tomato-labeled MSCs were suspended in 40 μl ECGM-MV2 media and 10 μl of Matrigel and transplanted subcutaneously to both hind limbs of each neonatal HA mouse at 2 weeks after birth. This age falls within the realm of neonatal age of mice and was necessary to properly perform the tail-clip assay. To minimize the possibility of intramuscular bleeding, a 28-G syringe was used for the subcutaneous injection.

### Bioluminescence imaging

Cells transplanted in the NSG mice were monitored via In Vivo Imaging Spectrum (IVIS) system (PerkinElmer) at designated time points. Animals were injected intraperitoneally with luciferase substrate D-luciferin (Gold Biotechnology) at 100 mg/kg body weight and maintained under anesthesia with 2% inhaled isoflurane for 5 min before imaging. The transplanted NSG mice were imaged at the day of transplantation and weekly or monthly thereafter up to 26 weeks after transplantation. The HA mice were imaged 7 days after transplantation. Images were analyzed by using Living Image®2.50 (Perkin Elmer). Total intensity was measured within a defined area of the signal. Baseline intensity was determined by using the same defined area where there is no positive signal in the same animal.

### In vitro co-culture of ECFCs and PMSCs

To study the effect of PMSCs on ECFCs, we designed direct and indirect co-culture of these two cell types. For direct co-culture, we combined 2.5 × 10^5^ GFP-labeled ECFCs with 2.5 × 10^5^ PMSCs and seeded them in a 100-mm tissue culture treated dish. For indirect co-culture, autoclaved silica gel that allowed media exchange was used to separate the ECFCs from PMSCs seeding the same number as in direct co-culture. 0.25 × 10^5^ ECFCs were seeded for ECFC only group. After 24 h of incubation in ECGM, we labeled proliferated cells with bromodeoxyuridine (BrdU) by culturing cells in 5 μM BrdU for additional 24 h. Cell cycle measurements were determined using the APC-conjugated BrdU Flor Kit (BD Biosciences) according to the manufacturer’s instruction. BrdU, an analog of the DNA precursor thymidine, can be incorporated into newly synthesized DNA by cells entering and progressing through the S phase of the cell cycle while 7-amino actinomycin D (7-AAD) is a fluorescent intercalator that undergoes a spectral shift upon association with DNA. With this combination, two-color flow cytometric analysis permits the enumeration and characterization of the cell cycle profile as well as apoptosis. We analyzed the data by gating the GFP-positive population to represent the ECFC.

To analyze the effect of PMSCs on ECFCs gene expression, 2.5 × 10^5^ ECFCs were seeded onto a 100-mm tissue culture treated dish overnight to allow them to attach on the plate. PMSCs that were treated with an irradiation dose of 10 Gy in order to inhibit proliferation were then added to the dish. PMSCs were allowed to be in contact with ECFCs for 0 min, 10 min, 6 h, and 24 h time points. At each time point, both the adhered and non-adhered cells were collected and total RNA was extracted using the RNeasyPlus Mini kit (Qiagen). Quantitative real-time polymerase chain reaction (qPCR) was performed by StepOnePlus real-time PCR system (Applied Biosystem, Waltham,MA) using SYBR Green PCR master mix (ThermoFisher Scientific) at an annealing temperature of 60 °C for 40 cycles. The primer sequences used are listed in Additional file 1: Table S1.

### Tail clip assay

A tail clip assay was performed to determine the blood loss in HA mice with or without receiving ECFCs and PMSCs. These mice were anesthetized using 2–3% isoflurane, and their tails were cut at 1.5 cm from the tip with a surgical blade. Following 5 min of free bleeding, pressure was applied to the cutting site for 1 min using a hemostat. Blood was collected into EDTA-coated tubes for 45 min after the cut. Coagulation efficacy was indicated by the amount of blood collected.

### Real-time PCR

To confirm the existence of human cells in the mouse tissue after transplantation, muscle tissue was harvested from animals that were sacrificed after IVIS imaging. A cube of muscle tissue about 0.5 cm × 0.5 cm × 0.5 cm in size with the strongest signal was collected from the site. Total DNA from 30 mg of homogenized tissue was extracted using DNeasy Blood & Tissue Kits (Qiagen). qPCR was performed by using TaqMan fast universal PCR master mix (ThermoFisher Scientific) at an annealing temperature of 60 °C for 40 cycles. The primer sequences used for human ERV3 and mouse GAPDH are listed in Additional file 1: Table S1. A standard curve was first generated for Ct value of each of the genes versus cell number. This curve was used to obtain the cell number based on the gene expression in our experimental tissues.

### Immunohistochemistry and immunocytochemistry

Muscle tissue samples that were obtained as described above were fixed with 4% paraformaldehyde for 24 h, protected by 30% sucrose dehydration for 48 h, and embedded in the O.C.T compound (Sakura Finetek USA). Serial sections were made at the thickness of 12 μm using a Cryostat (Leica CM3050S) and collected onto microscope slides (Matsunami Glass). Tissue sections were extensively washed with PBS, blocked with 5% BSA in PBS at room temperature for 1 h, and stained with primary antibody at 4 °C overnight. The dilutions of primary antibodies were rabbit anti-human B2M (Dako) 1:400, rabbit anti-mouse CD31 (Dako) 1:20, rabbit anti-SMA (Abcam) 1:50, goat anti-GFP (Novus Biologicals) 1:100, and sheep anti-FVIII (Affinity Biologicals) 1:100. Sections were incubated with their respective secondary antibodies diluted at 1:500 for 1 h at room temperature. The secondary antibodies were donkey anti-rabbit conjugated with Alexa647 (ThermoFisher Scientific), donkey anti-mouse conjugated with Alexa647 (ThermoFisher Scientific), donkey anti-goat (ThermoFisher Scientific) conjugated with Alexa488, and donkey anti-sheep conjugated with Alexa647 (ThermoFisher Scientific). The slides were counterstained with 1:5000 dilution of DAPI for 5 min, mounted with Prolong Diamond Antifade Mountant (Invitrogen), and imaged with a Zeiss Observer Z1 microscope.

To investigate the morphology change of ECFCs co-cultured with PMSCs, we seeded 1 × 10^4^ GFP-transduced ECFCs and 1 × 10^4^ Td-Tomato-transduced PMSCs on Matrigel-coated 24-well plate and cultured them in ECGM for 5 days. Cells were stained using 1:400 diluted rabbit anti-VE-Cadherin (Cell Signaling) or 1:40 diluted mouse anti-CD31 antibodies (Dako).

### Statistics

Data are reported as mean ± standard deviation (SD) for ELISA and chromogenic assay or mean ± standard error of mean (SEM) for bioluminescence image and tail clip assay. Statistical analysis of ELISA and chromogenic assay was performed by Student’s *t* test. Bioluminescence image analyses were performed using ANOVA with repeated measures. Tail clip assay analysis was performed by one-way ANOVA. All statistical analyses were performed using PRISM 7 (GraphPad Software Inc.), and differences were considered significant when *p* < 0.05.

## Results

### Cell isolation, characterization, and transduction

**Fig. 1:**
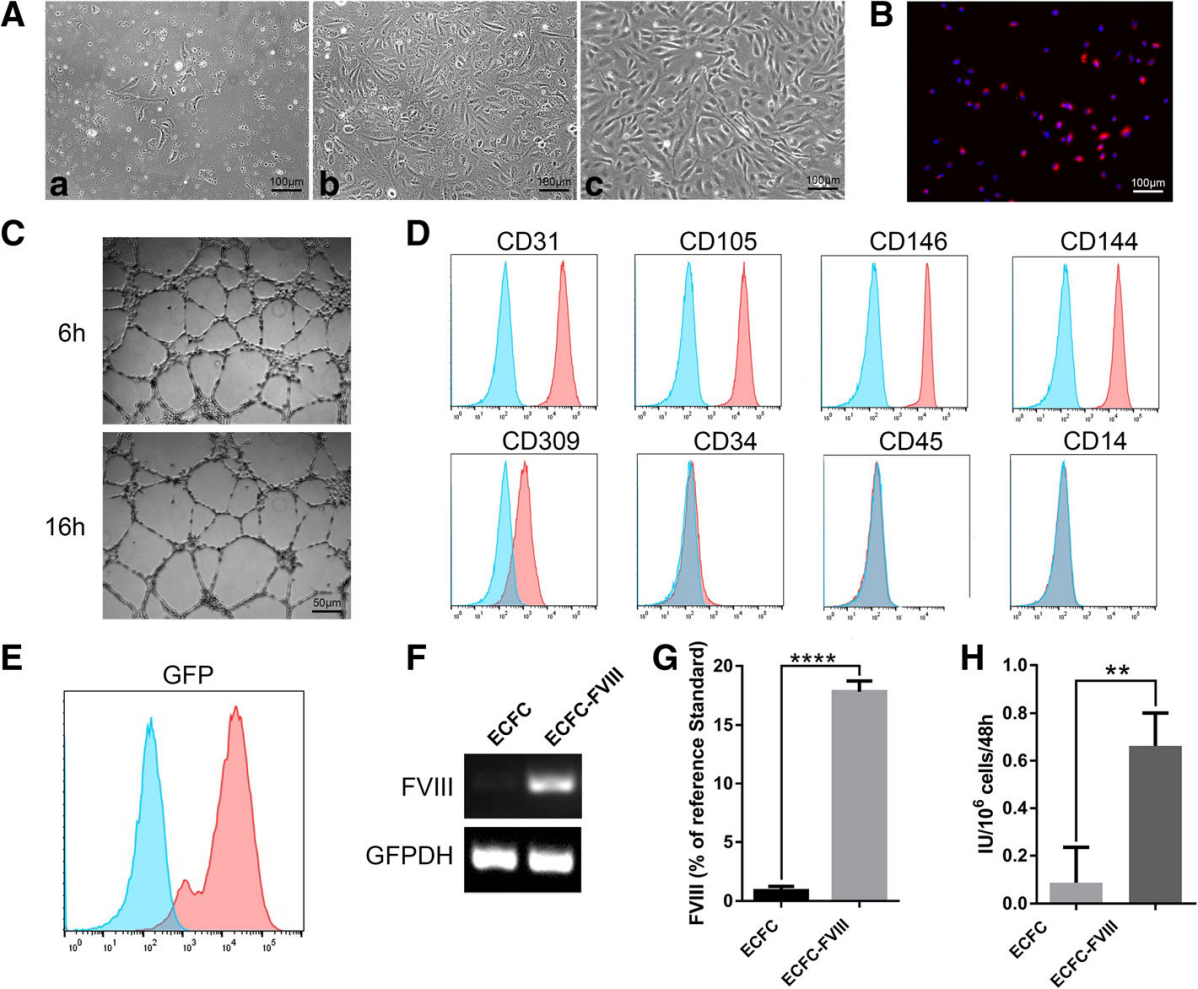
Characterization of cord blood-derived ECFCs before and after transduction. (A) Representative phase contrast images of isolated mononuclear cells from cord blood at day 3 (panel a) and day 7 (panel b) of in vitro culture. Typical cobblestone-like morphology of ECFCs at passage 6 is shown in (panel c). (B) Acetylated low-density lipoprotein uptake by ECFCs. (C) Tube formation by ECFCs on Matrigel. (D, E) Flow cytometric analyses of ECFC immunophenotype of surface markers (D) and GFP (E) expression after transduction (*n* = 1). Blue lines showed the negative controls, red lines showed positive expressions respectively. (F) Transduced and non-transduced ECFCs were characterized for BDD-FVIII gene expression by RT-PCR. (G) FVIII secretion detected by ELISA and (H) FVIII activity detected by chromogenic assay of ECFC culture media. Data are expressed as mean ± SD. ****p < 0.0001, **p < 0.01, *n* = 3

ECFCs were isolated from human cord blood using our previously established method [41]. The colonies formed by proliferating cells appeared as early as the third day of culture and gradually grew to confluency within 7–10 days of culture (Fig. 1A panels a, b). These cells showed a typical cobblestone-like morphology (Fig. 1A panel c) and their capability of acetylated low-density lipoprotein uptake and tube formation (Fig. 1B, C). Phenotypes of ECFCs were characterized by flow cytometry. They were positive for surface markers CD31 (99.96%), CD105 (99.83%), CD146 (99.96%), CD144 (99.86%), and CD309 (79.45%) and negative for CD34 (2.17%), CD45 (0.98%), and CD14 (0.27%) (Fig. 1D).

To overexpress FVIII in ECFCs, we transduced ECFCs with two lentiviral vectors. One was pCCLc-MNDU3-BDD-FVIII-PGK-NEO-WPRE encoding BDD-F8. The other one was pCCLc-MNDU3-LUC-PGK-EGFP-WPRE encoding luciferase and GFP for cell tracking and engraftment analyses. Transduction rate was 96.5% according to flow cytometry analysis of the GFP-positive cells (Fig. 1E). After viral transduction, ECFCs maintained endothelial immunophenotype and capability of acetylated low-density lipoprotein uptake and tube formation (Additional file 1: Figure S1A-C). RT-PCR analysis showed that BDD-F8 was highly expressed in the transduced ECFCs (ECFC-FVIII) but not in the non-transduced ECFCs (Fig. 1F). ELISA was used to measure the secreted amount of FVIII in the culture medium using human calibrator plasma as the reference standard. We found that the transduced ECFCs secreted markedly higher levels of BDD-FVIII (17.8 ± 1.08%/10^6^ cells) than the non-transduced cells (1.5 ± 0.12%/10^6^ cells) (Fig. 1G). The function of secreted BDD-FVIII was assessed by coagulation chromogenic assay. FVIII activity in the transduced cells (0.66 ± 0.13 IU/10^6^cells) was significantly higher than the non-transduced cells (0.09 ± 0.14 IU/10^6^cells) (Fig. 1H).

PMSCs were isolated and characterized as previously described [26] and transduced with a lentiviral vector pCCLc-MNDU3-F8-PGK-Tomato-WPRE. The characterization of the transduced PMSCs and Td-Tomato expression by flow cytometry are shown in Additional file 1: Figure S2 A-B. The transduction rate was 87.53%.

### In vivo engraftment of ECFCs and PMSCs in adult animals

**Fig. 2:**
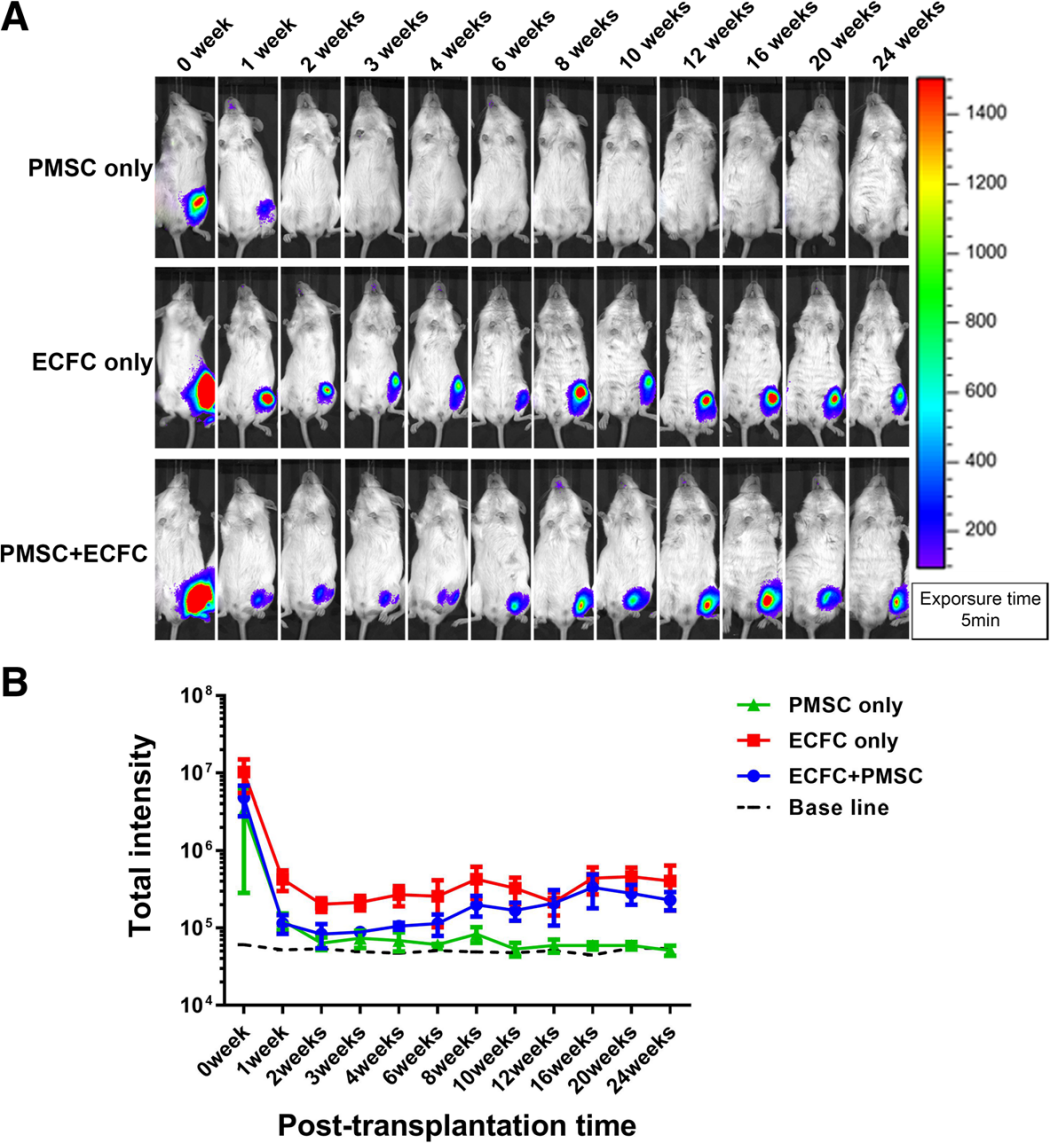
Cell retention in adult NSG mice. Adult NSG mice were transplanted with ECFCs or PMSCs alone or in combination. **a** Representative bioluminescence images of these mice in the indicated groups at different time points after cell transplantation. **b** The bioluminescence signals in different groups of mice were quantitatively analyzed. Data were expressed as mean ± SEM. *n* = 4

To investigate cell survival and engraftment of transplanted cells in adult animals, GFP/luciferin-labeled ECFCs (ECFC only group) or GFP/luciferin-labeled PMSCs (PMSC only group) or a mixture of Td-Tomato-labeled PMSCs plus GFP/luciferin-labeled ECFCs (co-transplantation group) were intramuscularly injected into the left hind limb of NSG mice at the adult age of 12 weeks. Cell survival and engraftment were monitored by IVIS. The bioluminescence signal in all the three groups decreased considerably during the first 3 weeks after transplantation. The signal in the PMSC only group reached baseline at the third week of post-transplantation and did not show any increase during the whole study period of 24 weeks. In contrast, the ECFC-only group and the ECFC/PMSC co-transplantation group also showed a decrease in the signal intensity in the first 2 weeks of post-transplantation but showed stable engraftment higher than the baseline during the whole time period up to 24 weeks of post-transplantation (Fig. 2).

### In vivo engraftment of ECFC and PMSC in neonatal animals

To investigate if the recipient age plays a role not only in cell survival and engraftment but also in the functional interaction between PMSCs and ECFCs, we designed another study in which PMSCs only, ECFCs only, or combination of ECFC and PMSC were transplanted by intramuscular injection into neonatal NSG mice at 3–5 days of age. The same experimental setting and numbers of cells were used in the neonatal mice as in the adult mice. The animals were monitored for 26 weeks post-transplantation.

**Fig. 3:**
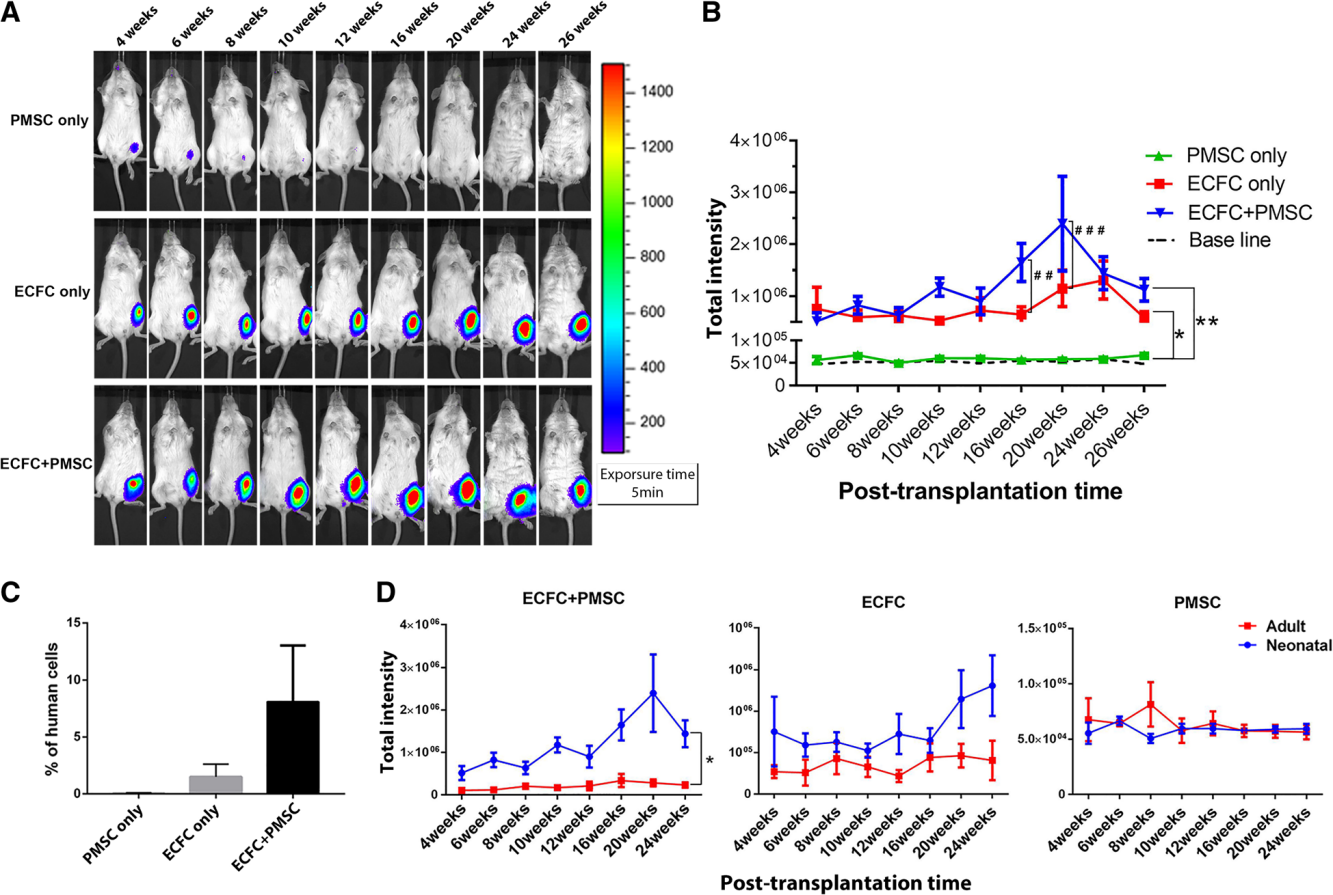
Long-term monitoring cell engraftment in mice transplanted at neonatal age. **a** Representative bioluminescence image of NSG mice transplanted with ECFCs only, PMSCs only or ECFCs plus PMSCs at 3–5 days after birth over 26 weeks post-transplantation. **b** Quantitative analysis of the bioluminescence signal intensity in these mice over time. **c** Real-time PCR analysis of different transplantation groups at 26 weeks post-transplantation for the presence of human cells using human-specific ERV3 and mouse GAPDH primers. (D) Comparison of cell retention at 4 to 24 weeks of post-transplantation of mice between transplanted cells at the neonatal period and adult. Data were expressed as mean ± SEM. *p < 0.05, **p < 0.01, ##p < 0.01, ###*p* < 0.001. *n* = 5

During the first 3 weeks of post-transplantation, the IVIS signal decreased ten times every week in all groups, indicating a decrease in cell survival during that period (Additional file 1: Figure S3A). No significant difference in cell retention among these three groups was detected during these first 3 weeks after transplantation using repeated measurement data variance analysis (Additional file 1: Figure S3B). The bioluminescence signal of the PMSC-only group continuously decreased and reached baseline after 12 weeks of transplantation (Fig. 3a top panel). In contrast, the bioluminescence signal in the ECFC-only group was maintained at the same intensity, markedly higher than the baseline throughout the 26-week period (Fig. 3a middle panel). In the co-transplantation group, the intensity was also maintained from 4- to 26-week period with the highest at the 20 weeks, suggesting long-term engraftment and proliferation of the transplanted cells (Fig. 3a bottom panel). The signal intensity was significantly higher in both the ECFC-only and co-transplantation group compared to the PMSC-only group. Further analysis of the difference between the ECFC-only group and the co-transplantation group, we performed Student’s *t* test for each time point and found that at 16 weeks and 20 weeks, the co-transplantation group had a significantly higher signal than ECFC-only group (*p* < 0.01 and *p* < 0.001) (Fig. 3b).

To quantitate the number of engrafted human cells in these animals, we performed real-time PCR analysis of the muscle tissues for human ERV3 expression and was normalized to mouse GAPDH expression. We found that 8.11 ± 4.93% (*n* = 3) of total number of cells are human cells in the co-transplanted group, while 1.50 ± 1.11% (*n* = 3) in the ECFC group. There was no detectable expression of human ERV3 in PMSC-only group (*n* = 3) (Fig. 3c).

We compared cell engraftment between the same time of mice that had been transplanted with ECFCs or combination of ECFCs and PMSCs at either the neonatal or the adult stage. In the period of 4 to 24 weeks of post-transplantation, the neonatal transplanted group had higher bioluminescence signal than the adult transplanted group in the co-transplanted group while in the ECFC-only and PMSC-only group, there is no significant difference (Fig. 3d). Our data suggest that performing cell transplantation with the combination of ECFC and PMSC in the neonatal period is superior to that in the adult period for long-term cell engraftment.

### Histological assessment of the transplanted cells

**Fig. 4:**
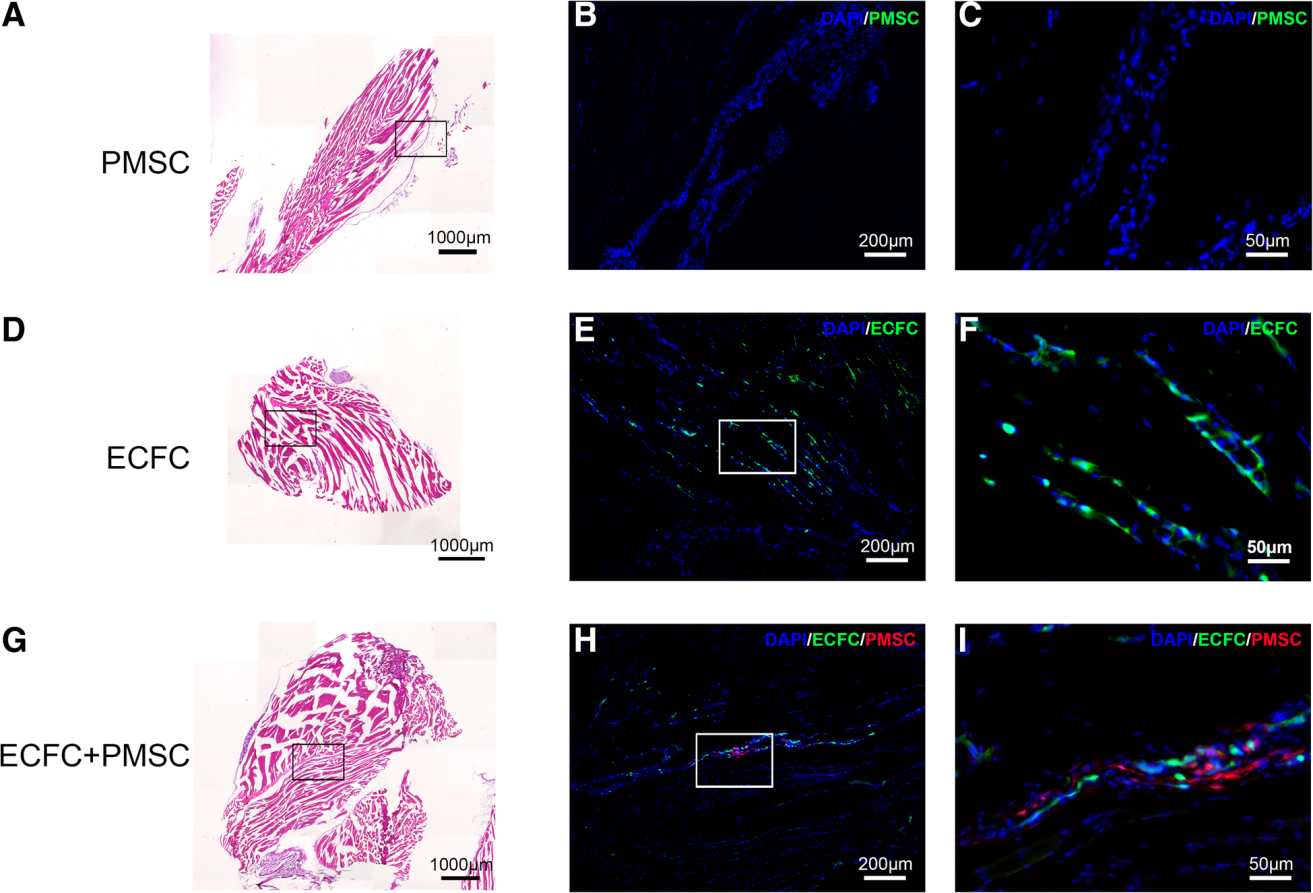
**a**–**i** Cell engraftment in the transplanted sites at 26 weeks post-transplantation. Representative images of the mouse tissues at the site of transplantation were collected based on their bioluminescence signal intensity. H&E stain is shown on the left panels. Fluorescence images are shown on the middle and the right panels with different magnifications. ECFC only and PMSC only groups are GFP (green) positive. In the co-transplanted group, ECFCs are GFP (green) positive and PMSCs are Td-Tomato (Red) positive

The mice that received cell transplantation at the neonatal stage were sacrificed at 26 weeks post-transplantation, and the muscle tissues at the site of injection were collected. Hematoxylin and eosin staining of these tissues showed the tissue structure and located the transplanted cells (Fig. 4a, d, g). The transplanted GFP-positive cells in the PMSC-only group were not detected (Fig. 4b, c) and ECFC-only group were detected under a fluorescence microscope (Fig. 4e, f). In the co-transplanted group, the GFP-positive ECFCs and Td-Tomato-positive PMSCs were also detected in adjacent area (Fig. 4h, i).

**Fig. 5:**
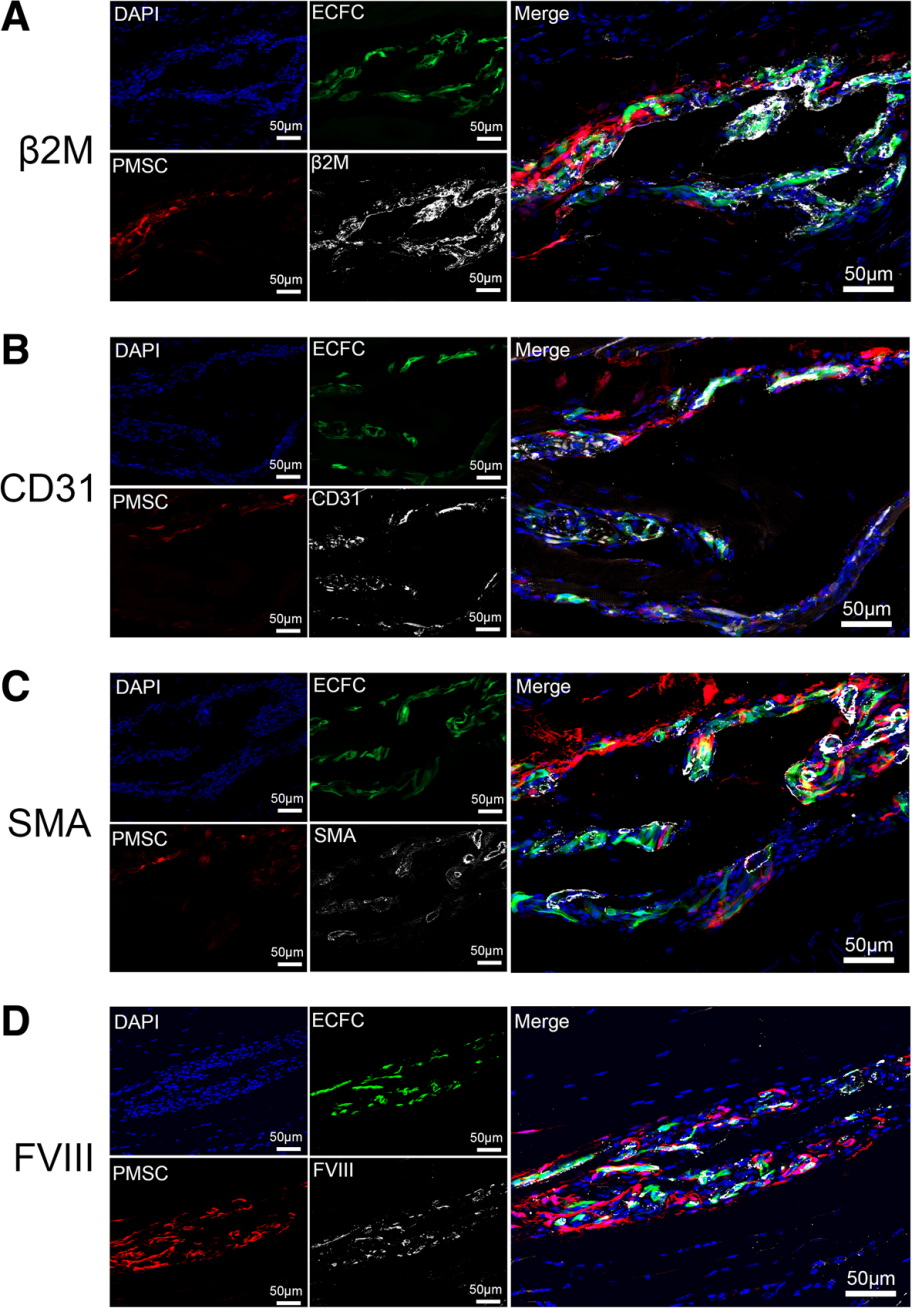
Characterization of the transplanted cells in the co-transplantation group. Representative immunohistochemistry staining images of the mouse tissue at the site of injection in the co-transplanted group. **a** Both ECFCs and PMSCs expressed β2M (white). **b** CD31 staining (white) was co-localized with ECFCs but not PMSCs. **c** SMA staining (white) was adjacent to ECFCs. **d** FVIII staining (white) was co-localized with ECFCs

Immunofluorescence staining was used for further characterization of the transplanted cells in the co-transplantation group. Human-specific beta-2 microglobulin (β2Μ) antibodies recognize a component of MHC class I molecules in human cells. We confirmed that both ECFCs and PMSCs expressed human β2M (Fig. 5a). Human CD31, an endothelial marker was present in ECFCs but not in PMSCs (Fig. 5b). Our data suggested that the transplanted ECFCs maintained their endothelial expression even at 26 weeks after transplantation.

Smooth muscle actin (SMA) expression was detected in tube structures in the denser area of ECFCs but not PMSCs, suggesting that ECFCs might be involved in the formation of functional blood vessels while PMSCs played a supporting function (Fig. 5c). In addition, both ECFCs and PMSCs expressed the FVIII protein due to both cell types were transduced with the FVIII vector (Fig. 5d).

### EndMT occurred during co-culture of ECFCs and PMSCs

**Fig. 6:**
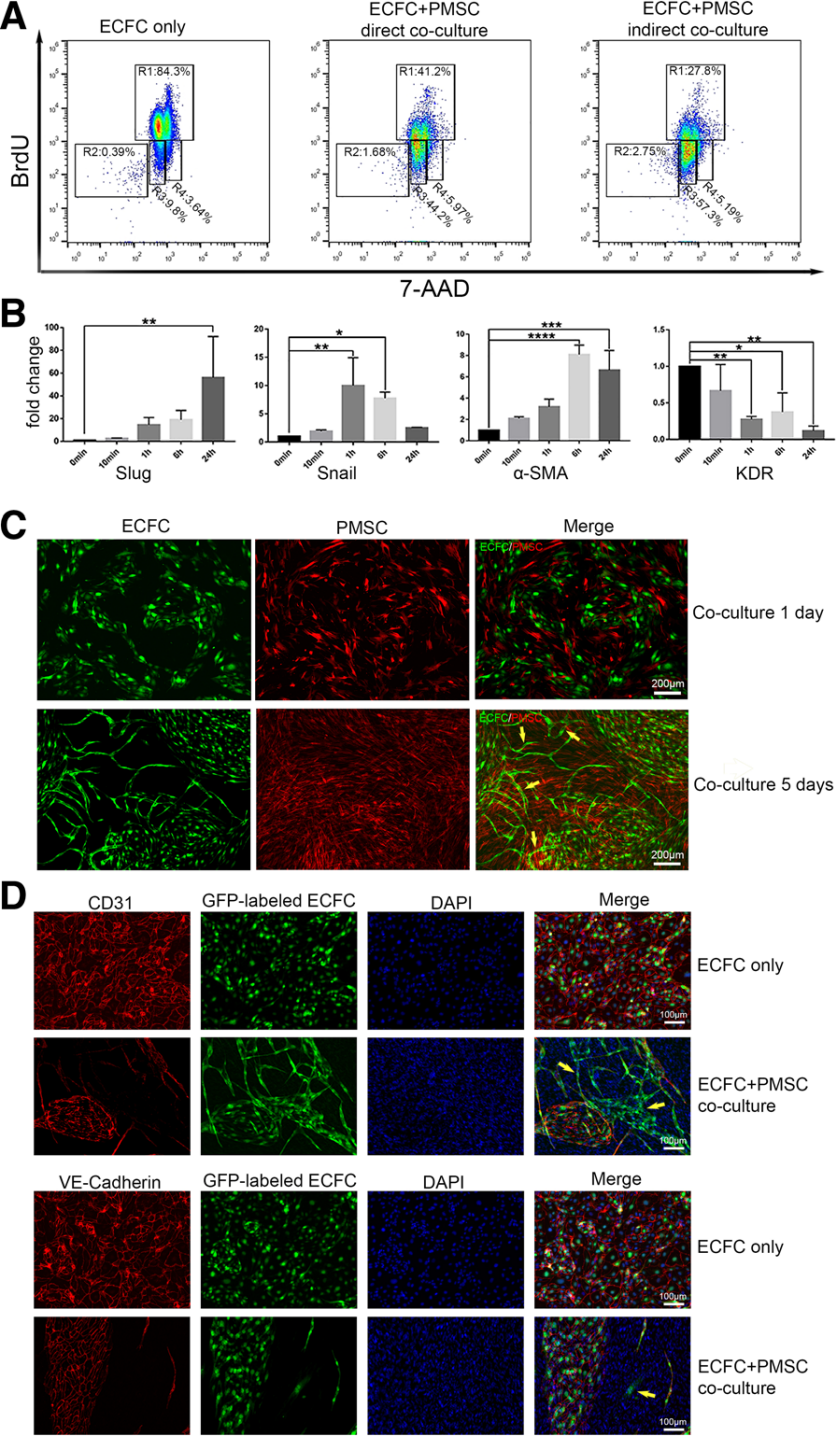
EndMT induced by co-culture ECFCs with PMSCs. **a** Cell cycle analysis by flow cytometry of ECFCs direct or indirect co-cultured with PMSCs. R1: S phase, R2: apoptotic, R3: G0/G1 phase, R4: G2+M phase. **b** Real-time PCR of EndMT-related gene. Data are expressed as mean ± SD. ***p < 0.001, **p < 0.01, *p < 0.05, *n* = 3. **c** Cell phenotype of co-cultured ECFCs and PMSCs. **d** Immunocytochemistry staining image of 5 days co-cultured ECFCs and PMSCs

To investigate the mechanisms of how PMSCs help ECFC engraftment, we performed cell cycle analysis by labeling co-cultured ECFCs and PMSCs with BrdU and 7-AAD. After 24 h of co-culture, the S phase (BrdU incorporation) population in ECFCs alone was 84.3% but was decreased to 41.2% when ECFCs were directly co-cultured with PMSCs and 27.8% when they were indirectly co-cultured with PMSCs. At the same time, the G0/G1 population in ECFCs alone was 9.8% but was increased to 44.2% in ECFCs when they were directly co-cultured with PMSCs and 57.3% when they were indirectly co-cultured with PMSCs. (Fig. 6a). Our data show that PMSCs induced growth arrest of ECFCs in vitro, regardless of direct or indirect contact.

Endothelial-to-mesenchymal transition (EndMT) is a process by which endothelial cells lose their cell-specific markers and morphology and acquire a mesenchymal cell-like phenotype [42]. Recently, EndMT has been found to play a key role in the early stages of angiogenesis [43]. We investigate whether PMSCs promote EndMT in ECFCs. We quantified the expression of two transcription factors Slug and Snail that are implicated in EndMT. Both factors were activated in ECFCs after co-cultured with PMSCs. Also, the expression of α-SMA was upregulated, and expression of KDR was downregulated implicating the loss of an EC phenotype leading to a MSC-like phenotype (Fig. 6b). After 5 days of co-cultivation, a portion of the ECFCs was no longer a cobblestone-like phenotype and formed tubular structures with the help of PMSCs (Fig. 6c pointed by the arrow). Therefore, long-term co-culture is likely to ultimately contribute to angiogenesis. Immunocytochemical staining indicated that some ECFCs lost the expression of CD31 or VE-Cadherin (Fig. 6d pointed by the arrow) after co-culture, demonstrating EndMT.

### Attenuation of the bleeding symptom of HA mice by co-transplantation of ECFCs and PMSCs

**Fig. 7:**
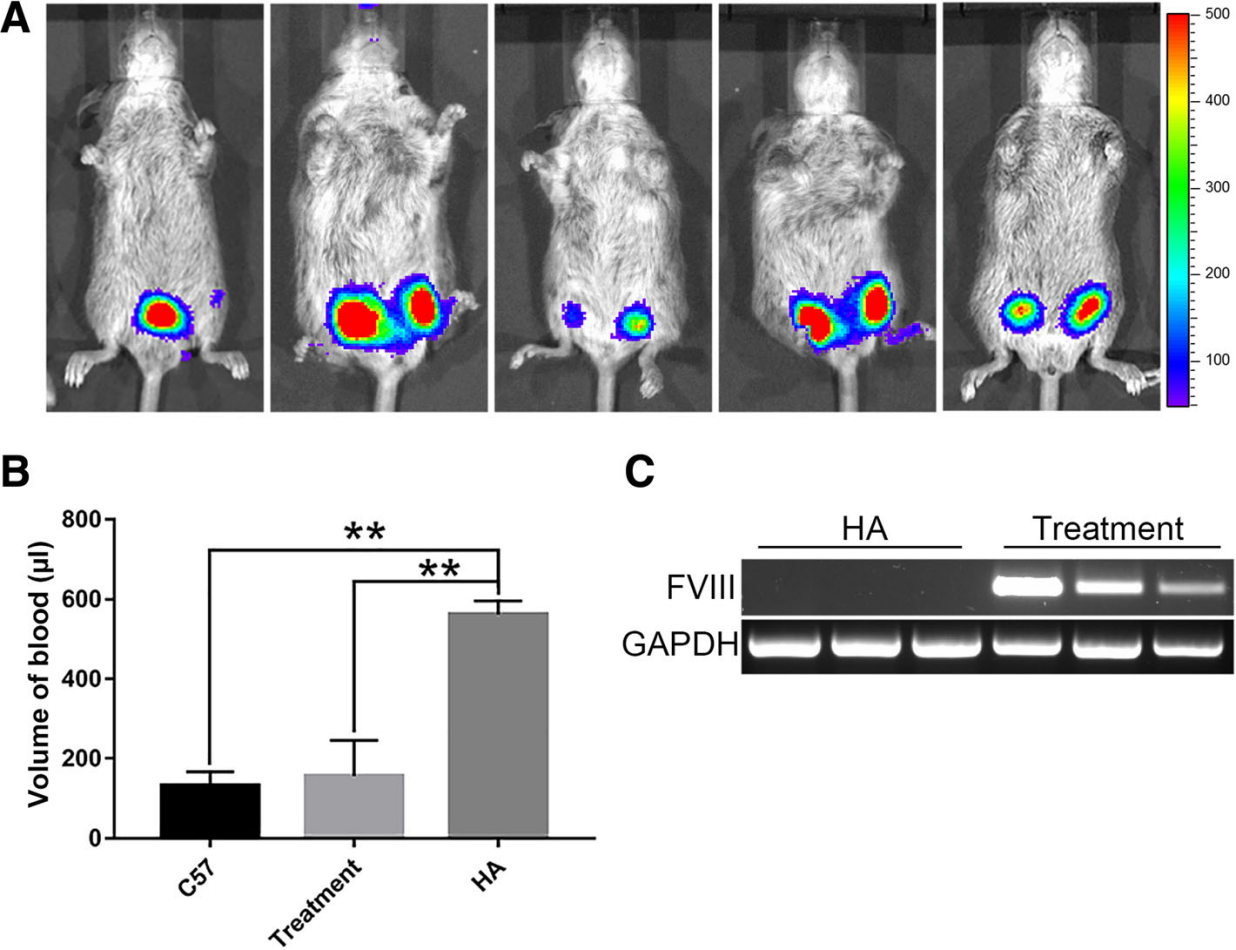
Phenotype correction of hemophilia A mice by co-transplantation of ECFCs and PMSCs. **a** Bioluminescence images of the HA mice 7 days after co-transplantation of ECFCs and PMSCs. **b** The volume of blood loss in a tail clip assay of C57BL/6 mice, HA mice, and the HA mice transplanted with ECFCs and PMSCs. Data were expressed as mean ± standard error. *n* = 4 of the C57 group, *n* = 5 of treatment group, *n* = 3 of HA group. **p < 0.01. **c** RT-PCR analysis of F8 expression in the limb tissues of HA mice and the HA mice transplanted with ECFCs and PMSCs

In order to evaluate whether co-transplantation of ECFCs and PMSCs has therapeutic effect on treating hemophilia A, we subcutaneously transplanted 3 × 10^6^ ECFCs and 2 × 10^6^ PMSCs into the left and right hind limbs of each neonatal HA mice at 2 weeks after birth. One week post-transplantation, bioluminescence imaging showed that the transplanted cells were retained in all five HA mice (Fig. 7a). A tail clip assay was performed to detect blood loss in these treated mice and was compared to control non-treated HA mice and control normal C57BL/6 mice. The blood loss volume in the control C57BL/6 mice and in the control HA mice was 131.5 ± 13.3 μl and 562.13 ± 19.84 μl, respectively (Fig. 7b). The blood loss volume in the HA mice co-transplanted with ECFCs and PMSCs was 155.78 ± 44.93 μl, which is similar to the normal C57BL/6 mice and is significantly less than that of the control HA mice (Fig. 7b). RT-PCR analysis further confirmed the expression of *F8* in the mouse tissues at the site of injection, while the control HA mice had no detectable expression of *F8* (Fig. 7c). Our data demonstrated that co-transplantation of ECFCs and PMSCs significantly attenuated the bleeding symptom of HA mice.

## Discussion

During the last decade, numerous attempts have been made to develop a long-term cure for monogenic disorders like hemophilia A. For hemophilia A treatment, increasing circulating clotting FVIII level to above 1% of normal can significantly reduce risks of spontaneous internal bleeding [44]. The primary cellular source of FVIII biosyntheses has been controversial for a long time. Liver transplantation studies in the 1960s and 1980s have shown that liver is the major source of FVIII [45, 46]. Although earlier evidence has suggested hepatocytes to be the sole source of FVIII expression in the liver [47], it was later proven to be primarily the LSECs [12, 13, 48]. In addition to liver, it was shown that endothelial cells from other organs like lung, heart, intestine, and skin also produce FVIII [49]. Therefore, using endothelial cells seems to be a suitable candidate for cell-mediated gene therapy for HA.

ECFCs are a group of cells with high proliferation capacity. They are rare cells found at a concentration of about 0.05–0.2 cells/ml in adult peripheral blood but are highly abundant in human umbilical cord blood at a concentration of about 2–5 cells/ml [50]. Numerous studies have shown that ECFCs obtained from cord blood are less mature with high proliferative potential in vitro and in vivo than those obtained from adult bone marrow [51]. Hence, cord blood could be a better source of ECFCs than bone marrow. Consistent with previous studies [52–54], we showed that the cord blood-derived ECFCs expressed endothelial cell-surface antigens CD31, CD105, CD144, CD146, and CD309 and did not express the hematopoietic or monocyte cell surface antigens CD14, CD45, or CD34. Their endothelial functional phenotype was demonstrated by their ability to incorporate Ac-LDL and to form tubes when seeded on matrigel. There is a controversy on whether EPC expresses FVIII. Campioni et al. reported that EPCs from adult peripheral blood express FVIII according to the ICC staining [55]. However, Christian et al. reported that no FVIII protein could be detected by Western blot in concentrated supernatants of un-transduced cord blood derived endothelial cells (CBECs) [56]. The discrepancy and inconsistency are likely due to different sources and/or stages of EPCs/ECFCs (neonatal vs adult) were assessed in these studies. In consistent with the latter report, our data showed that cord blood-derived ECFCs did not express an appreciable amount of FVIII. To increase the amount of FVIII secretion, we transduced these cells with lentivirus to overexpress FVIII.

To improve the engraftment of transplanted ECFCs, co-transplantation of two or more types of cells has been considered. Previous researches have shown that co-transplantation of ECFCs with MSCs improves vascularization and engraftment compared to ECFCs alone. MSCs efficiently stabilized nascent blood vessels in vivo by functioning as perivascular precursor cells [57–59]. Co-transplantation of ECFCs and MSCs significantly promote tissue recovery in cardiovascular disease, cerebrovascular disease, and during bone regeneration [60]. We are the first, to our knowledge, to investigate co-transplantation of ECFCs and PMSCs to treat hemophilia A. In our study, we found that ECFCs showed persistent engraftment when transplanted alone or along with PMSCs in immunodeficient mice. MSCs significantly enhanced ECFCs engraftment at the later time points of post-transplantation. Given the unique immunomodulatory properties of PMSCs, we expect that this co-transplantation strategy could yield even more beneficial results in the immune competent clinical setting. Importantly, significant attenuation of the bleeding phenotype of the HA mice was achieved by co-transplantation of ECFCs and PMSCs. However, we could not detect FVIII by ELISA in the blood collected during the tail-clip assay. This might be due to the low circulating levels of FVIII in the plasma in the transplanted HA mice. In addition, since ELISA was performed to the blood samples collected during the tail-clip assay, some circulating FVIII could have already been consumed during coagulation at the site of the wound. Future experiments will involve optimizing the detection method of FVIII, improving approaches to increase FVIII concentration in the blood, in addition to using different sets of animals for the measurements of plasma FVIII levels and for the tail clip assay.

The timing of the gene and cell therapy for an HA patient plays a significant role in determining outcomes. Neonatal gene therapy is a promising strategy for treating multiple congenital diseases that can be diagnosed shortly after birth. Therapeutic gene expression early in life may prevent the development of irreversible damage caused by the disease. In addition, introducing a non-preexisting expression of the therapeutic protein prior to maturation of immunity may enable immune tolerance towards the therapeutic protein [61, 62]. This is especially relevant to HA because about one third of the HA patients that received FVIII replacement therapy developed antibody inhibitors against FVIII. Our data showed that cell transplantation at the neonatal stage resulted in a higher level of engraftment compared to cell transplantation at the adult stage. We demonstrated further that co-transplantation of ECFCs with PMSCs into the neonatal HA mice functionally alleviated their bleeding symptom. Our study provides proof-of-concept that neonatal cell-based gene therapy is effective and preferable in treating hemophilia A.

The mechanism of interaction between PMSCs and ECFCs during co-transplantation is not completely understood. In our study, we showed that PMSCs induced growth arrest of ECFCs in vitro and this might potentially have an effect on the survival of the cells during our co-transplantation studies. In addition, we found that some of ECFCs developed EndMT after co-culture with PMSCs, which is probably one of the mechanisms by which MSCs increase the rate of ECFC colonization. EndMT was originally discovered as an important mechanism of cardiac development [63] and has been widely used in many studies to evaluate several systemic disease processes such as fibrosis and tumors [64–66]. But recent studies have shifted attention to the physiological rather than pathological phenomena of this mechanism. Previous reports have shown that vascular supporting cells, such as pericytes and/or smooth muscle cells, may be derived from endothelial cells themselves; therefore, EndMT may be an important mechanism for recruiting such parietal cells during angiogenesis [43, 67]. EndMT can be induced by fibroblast growth factor 1 (FGF-1) via proteolytic matrix metalloproteinases 1 (MMP-1) activity and plays a key role in early-stage angiogenesis [68]. EndMT has been reported to be an important mechanism underlying neointimal formation in interpositional vein graft via TGF-β–Smad2/3–Slug signaling pathway [42]. These findings indicate that EndMT is likely to have important implications for cell migration and ultimately function under physiological conditions.

PMSCs and ECFCs may interact with each other through paracrine mechanism. Several studies have suggested paracrine mechanism of MSCs in angiogenesis [69, 70]. NOTCH signaling was shown to contribute to mesenchymal priming of ECFCs by stimulating a more differentiated endothelial phenotype [39, 71, 72]. Interestingly, we found that PMSCs survived persistently for more than 6 months when transplanted together with ECFCs, while the PMSCs transplanted alone could rarely survive after 8 weeks post-transplantation. It has been shown that ECFCs can function as paracrine mediators to support MSCs by providing PDGF-BB, FGF-2, and other critical angiogenic factors [38, 73], that in turn promote the expansion of MSCs and are essential for the proper functioning of various stem cell niches [74–76]. But, these studies did not address the long-term engraftment rate of the co-transplantation. Our study suggests a mutually beneficial effect between ECFCs and MSCs with respect to their long-term cell engraftment. In summary, our study demonstrated that co-transplantation of ECFCs with PMSCs at the neonatal age can achieve stable, long-term engraftment. Therefore, the strategy of co-transplantation of ECFCs with PMSCs holds great promise for the treatment of HA.

## Conclusions

HA is an ideal target for cell-based gene therapy, but successful treatment has been hampered by insufficient long-term engraftment of the therapeutic cells. We co-transplanted PMSCs and ECFCs into neonatal NSG mice which achieved a stable engraftment over 26 weeks and reduced the blood loss volume of F8 knock-out mice in a tail-clip assay. This work demonstrated that co-transplantation of ECFCs with PMSCs at the neonatal age is a potential strategy to achieve stable, long-term engraftment, and thus holds great promise for cell-based treatment of HA.

## Additional file

Additional file 1: **Table S1.** (1) Primer sequence of RT-PCR. (2) Primer sequence of EndMT. (3) Primer sequence of Real-time PCR. **Figure S1.** Characterization of the transduced cord blood ECFCs. **Figure S2.** Characterization of the transduced PMSCs. **Figure S3.** Short-term monitoring of cell retention after transplantation in neonatal mice. (DOCX 786 kb)

## Abbreviations

7-AAD: 7-Amino actinomycin D
AAV5: Adeno-associated virus serotype 5
BDD-FVIII: B domain deleted factor VIII
BM-MSCs: Marrow-derived mesenchymal stromal cells
BrdU: Bromodeoxyuridine
ECFCs: Endothelial colony-forming cells
ECs: Endothelial cells
ELISA: Enzyme-linked immunosorbent assay
EndMT: Endothelial-to-mesenchymal transition
EPCs: Endothelial progenitor cells
FGF-1: Fibroblast growth factor 1
FGF-2: Fibroblast growth factor 2
FVIII: Factor VIII
GFP: Green fluorescence protein
HA: Hemophilia A
HGF: Hepatocyte growth factor
IVIS: In Vivo Imaging Spectrum
LSECs: Liver sinusoidal endothelial cells
MCT: Monocrotaline
MMP-1: Matrix metalloproteinases 1
MNCs: Mononuclear cells
MOI: Multiplicity of infection
PDGF-BB: Platelet-derived growth factor-BB
PMSCs: Placenta-derived mesenchymal stromal cells
PST: Protein substitution therapy
qPCR: Quantitative real-time polymerase chain reaction
SD: Standard deviation
SEM: Standard error of mean
SMA: Smooth muscle actin
VEGF: Vascular endothelial growth factor
β2Μ: Beta-2 microglobulin
