# Supplementary material for: Potential long-term treatment of hemophilia A by neonatal co-transplantation of cord blood-derived endothelial colony-forming cells and placental mesenchymal stromal cells
Source: Stem Cell Res Ther. 2019 Jan 22;10:34. doi: 10.1186/s13287-019-1138-8 (PMC6341603; doi:10.1186/s13287-019-1138-8)
Supplement: Supplementary file 1 — Table S1. (1) Primer sequence of RT-PCR. (2) Primer sequence of EndMT. (3) Primer sequence of Real-time PCR. Figure S1. Characterization of the transduced cord blood ECFCs. Figure S2. Characterization of the transduced PMSCs. Figure S3. Short-term monitoring of cell retention after transplantation in neonatal mice. (DOCX 786 kb) [file 13287_2019_1138_MOESM1_ESM.docx]

**Additional file 1**

*Table S1-1: Primer sequence of RT-PCR*

| Gene | Sequence | |
| --- | --- | --- |
| human GAPDH | Forward | 5’ gccagcatcgccccacttga 3’ |
|  | Reverse | 5’ cggtcgtagcggggtgaact 3’ |
| BBD-F8 | Forward | 5’ cagtcttgaaacgccatcaa 3’ |
|  | Reverse | 5’ aatcccagagcctctccact 3’ |

*Table S1-2: Primer sequence of EndMT*

| Gene | Sequence | |
| --- | --- | --- |
| Slug | Forward | 5’ actccgaagccaaatgacaa 3’ |
|  | Reverse | 5’ ctctctctgtgggtgtgtgt 3’ |
| Snail | Forward | 5’ ggcaatttaacaatgtctgaaaagg 3’ |
|  | Reverse | 5’ gaatagttctgggagacacatcg 3’ |
| α-SMA | Forward | 5’ aagcacagagcaaaagaggaat 3’ |
|  | Reverse | 5’ atgtcgtcccagttggtgat 3’ |
| KDR | Forward | 5’ gtggcacccacgatcaca 3’ |
|  | Reverse | 5’ gacttcgatgctttccccaat 3’ |
| Human GAPDH | Forward | 5’ gagtcaacggatttggtcgt 3’ |
|  | Reverse | 5’ ttgattttggagggatctcg 3’ |

*Table S1-3: Primer sequence of Real-time PCR*

| Gene | Sequence | |
| --- | --- | --- |
| human ERV3 | Probe | 5’ 6FAM-tcttccctcgaacctgcaccatcaagtca-TAMRA 3’ |
|  | Forward | 5’ catgggaagcaagggaactaatg 3’ |
|  | Reverse | 5’ cccagcgagcaatacagaattt 3’ |
| mouse GAPDH | Probe | 5’ 6FAM-tcagcaatgcatcatcctgcaccaccaact-TAMRA 3’ |
|  | Forward | 5’ accacgagaaatatgacaactca 3’ |
|  | Reverse | 5’ cccactgcctacataccatgagc 3’ |


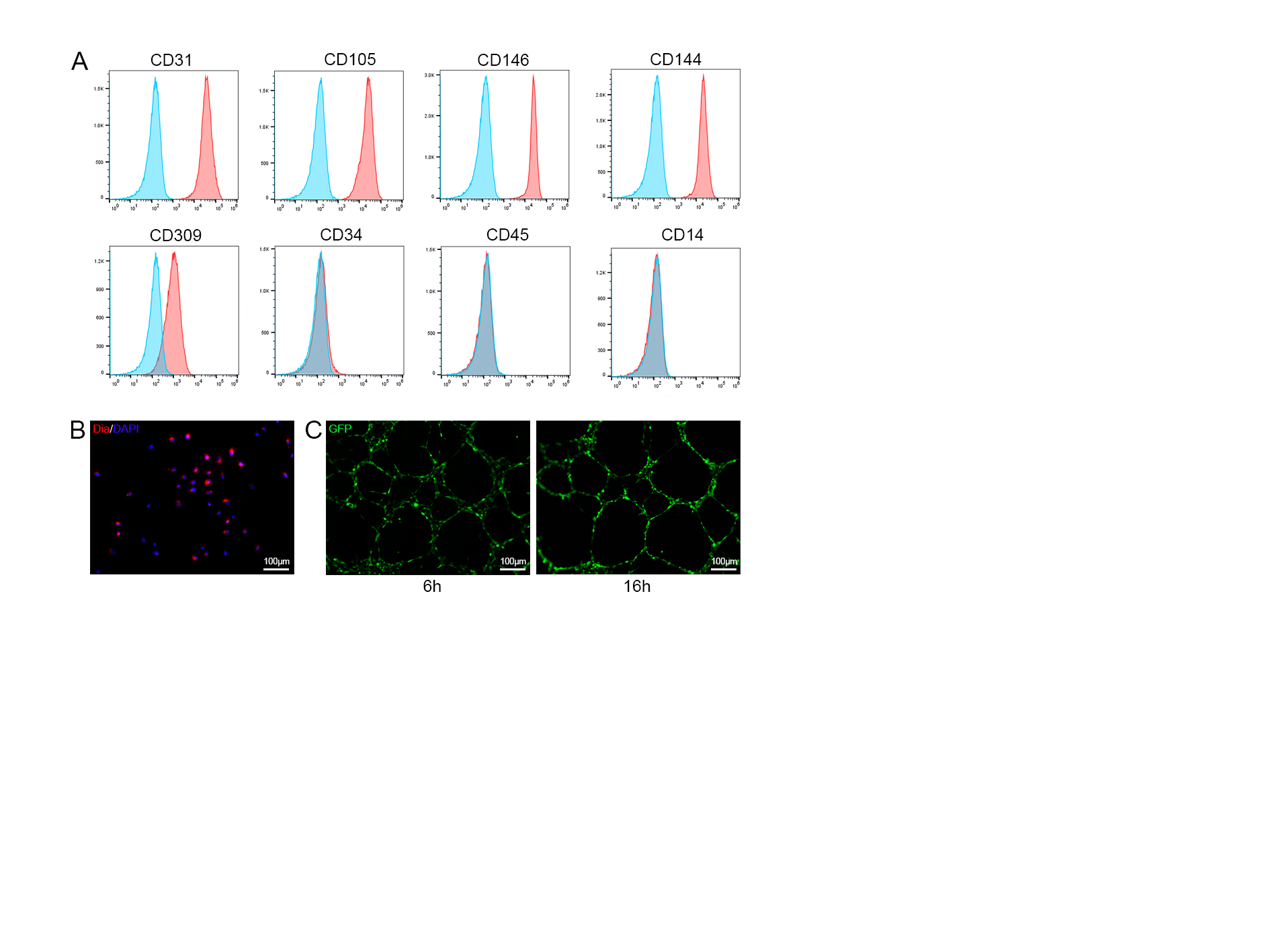


*Figure S1: Characterization of the transduced cord blood ECFCs*

*(A) Flow cytometric analysis of ECFC immunophenotype for expression of surface markers. (B) Acetylated low-density lipoprotein uptake by ECFCs. (C) Matrigel tube formation by ECFCs.*

*
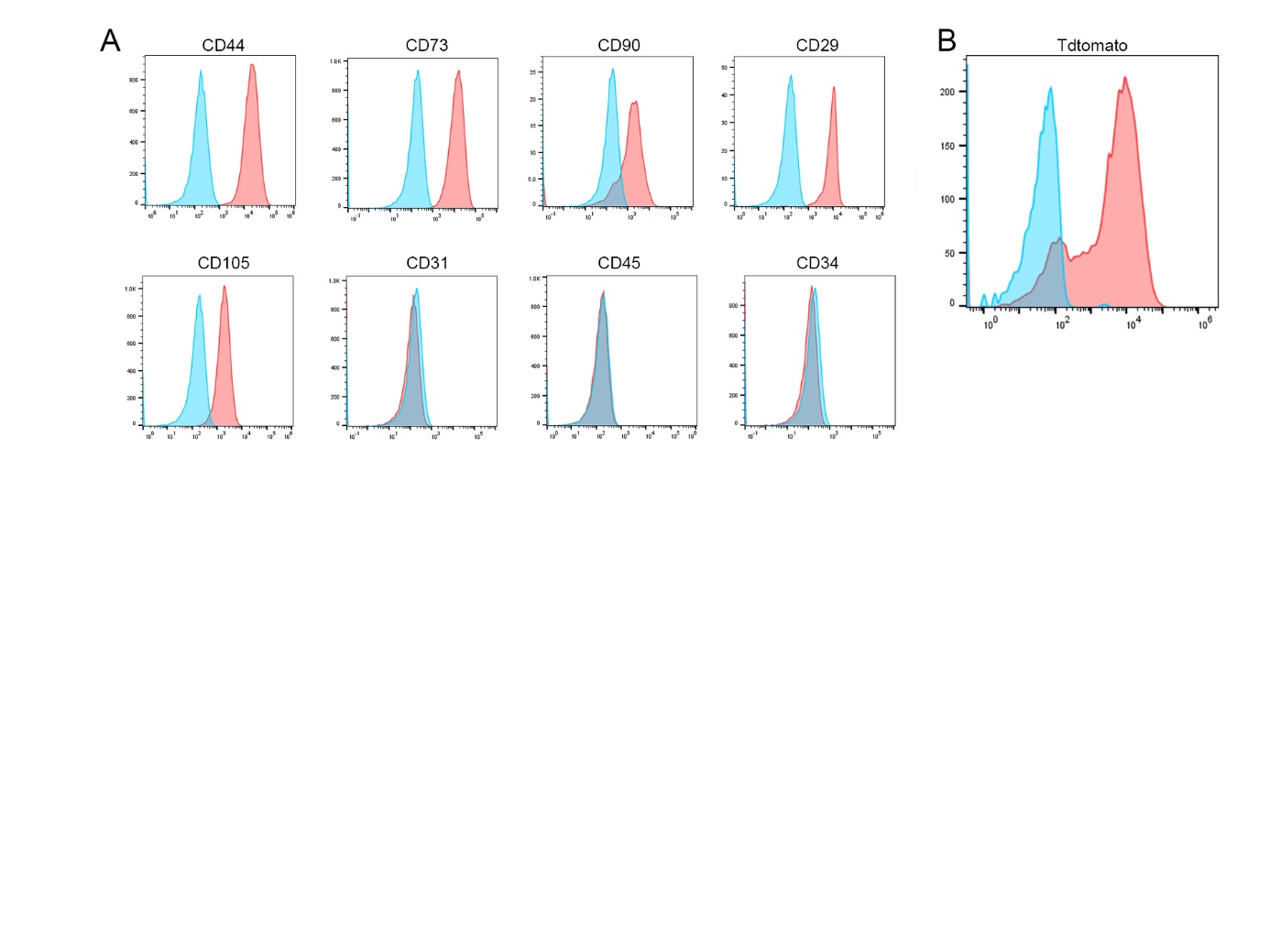
Figure S2: Characterization of the transduced PMSCs*

*Flow cytometric analysis of immunophenotype for expression of surface markers in transduced PMSCs (A) and transduction rate was characterized by Td-Tomato expression rate (B).*

*
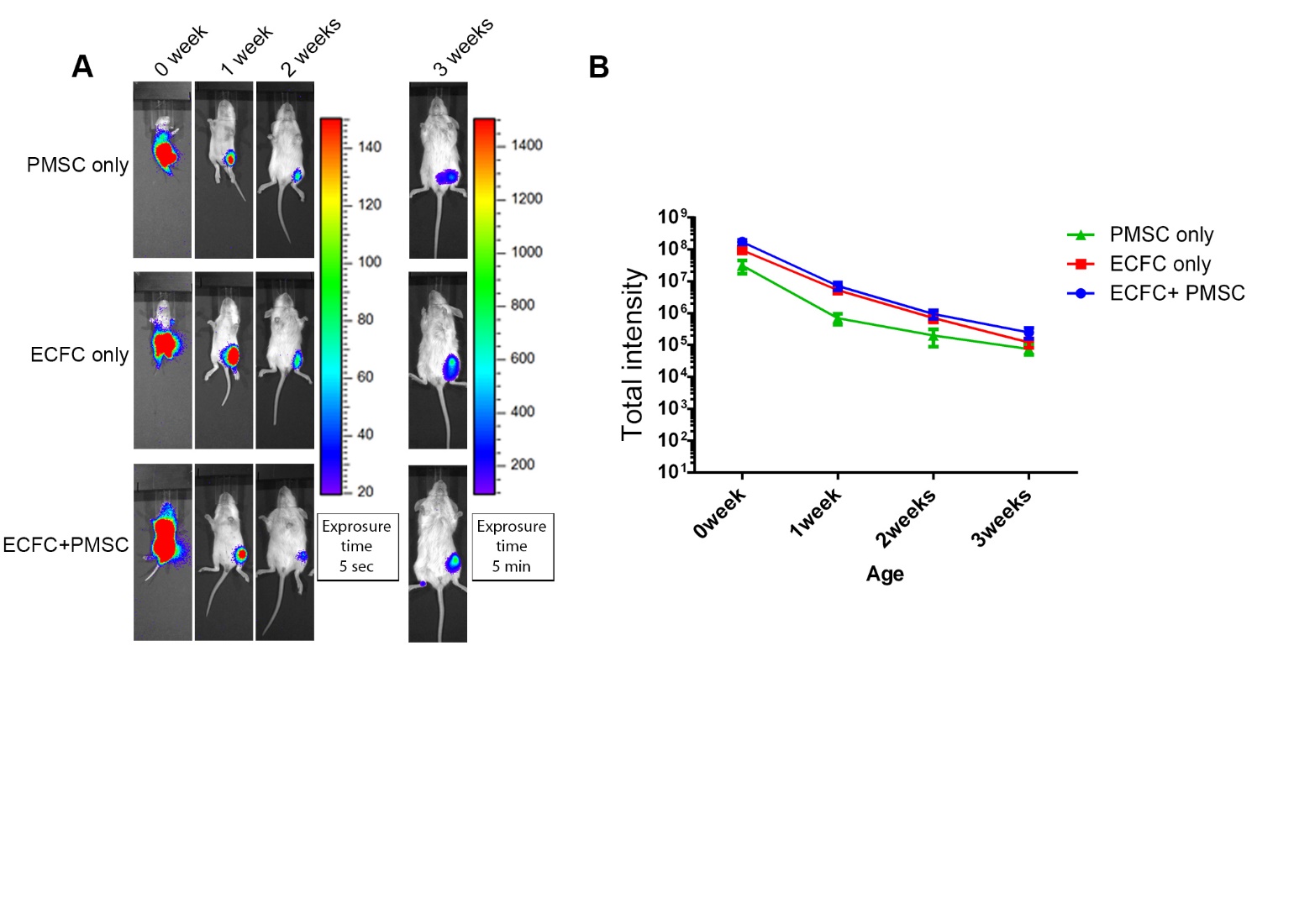
Figure S3: Short-term monitoring of cell retention after transplantation in neonatal mice*

*(A) Representative bioluminescence images of neonatal transplanted mice at different time points after transplantation with ECFCs or PMSCs or combination of ECFCs and PMSCs. (B) Quantitative analysis of the Bioluminescence intensity at 0 to 3 weeks in these mice.* *Variance analysis showed that the IVIS signal significantly decreased by time in all three groups(p<0.001), but there was no significant difference between these three groups using ANOVA by repeated measures. Data were expressed as mean ± SEM, n=5.*
